# Automated lead toxicity prediction using computational modelling framework

**DOI:** 10.1007/s13755-023-00257-4

**Published:** 2023-11-20

**Authors:** Priyanka Chaurasia, Sally I. McClean, Abbas Ali Mahdi, Pratheepan Yogarajah, Jamal Akhtar Ansari, Shipra Kunwar, Mohammad Kaleem Ahmad

**Affiliations:** 1 School of Computing, Engineering & Intelligent Systems, Ulster University, Derry, Londonderry, BT487JL UK; 2 School of Computing, Ulster University, Co. Antrim, Newtownabbey, BT370QB UK; 3 Department of Biochemistry, King George’s Medical University, Lucknow, Uttar Pradesh 226003 India; 4 Department of Obstetrics & Gynecology, Faculty of Medicine, Era University, Lucknow, Uttar Pradesh 226003 India

**Keywords:** Lead toxicity, Data analytics, Prediction modelling, Maternal lead exposure, Boruta algorithm, Sociodemographic, Machine learning

## Abstract

**Background:**

Lead, an environmental toxicant, accounts for 0.6% of the global burden of disease, with the highest burden in developing countries. Lead poisoning is very much preventable with adequate and timely action. Therefore, it is important to identify factors that contribute to maternal BLL and minimise them to reduce the transfer to the foetus. Literacy and awareness related to its impact are low and the clinical establishment for biological monitoring of blood lead level (BLL) is low, costly, and time-consuming. A significant contribution to an infant’s BLL load is caused by maternal lead transfer during pregnancy. This acts as the first pathway to the infant’s lead exposure. The social and demographic information that includes lifestyle and environmental factors are key to maternal lead exposure.

**Results:**

We propose a novel approach to build a computational model framework that can predict lead toxicity levels in maternal blood using a set of sociodemographic features. To illustrate our proposed approach, maternal data comprising socio-demographic features and blood samples from the pregnant woman is collected, analysed, and modelled. The computational model is built that learns from the maternal data and then predicts lead level in a pregnant woman using a set of questionnaires that relate to the maternal’s social and demographic information as the first point of testing. The range of features identified in the built models can estimate the underlying function and provide an understanding of the toxicity level. Following feature selection methods, the 12-feature set obtained from the Boruta algorithm gave better prediction results (*k*NN = 76.84%, DT = 74.70%, and NN = 73.99%).

**Conclusion:**

The built prediction model can be beneficial in improving the point of care and hence reducing the cost and the risk involved. It is envisaged that in future, the proposed methodology will become a part of a screening process to assist healthcare experts at the point of evaluating the lead toxicity level in pregnant women. Women screened positive could be given a range of facilities including preliminary counselling to being referred to the health centre for further diagnosis. Steps could be taken to reduce maternal lead exposure; hence, it could also be possible to mitigate the infant’s lead exposure by reducing transfer from the pregnant woman.

## Introduction

Lead, a universal pollutant, has been traced in every aspect of environmental and biological systems [1]. The global death rate attributable to high BLL has increased steadily by 21% [2]. In 2019, lead poisoning is attributed to more than 900,000 premature deaths globally (1.6% of overall deaths) and this is similar to the number of deaths caused by HIV/AIDs [3]. To reduce the consequences of lead exposure, UNICEF and Pure Earth recommend that countries should strengthen health systems by including more appropriate detection, monitoring, and reporting approaches [1]. Lead abatement measures have been adopted in most countries in recent years, including the removal of lead from petrol and paint. This has resulted in a lead concentration decline in children and adults [4]. Nevertheless, lead poisoning is still an unrecognised or partially recognised problem, with no to very limited, ineffective screening and prevention programmes. It has been reported that lead persists above the permissible limit allowed in many developing countries disease [5–7].

Infants and children are specifically at risk of lead exposure. Recent ground-breaking research from UNICEF discovered that 1 in 3 children (i.e., around 800 million) worldwide have BLL at or above 5 μg/dL, a level that the Centres for Disease Control and Prevention (CDC) has urged to initiate action [8]. It has been reported by the WHO that even low lead level in children is related to learning difficulties, behavioural issues, and decreased intelligence [8]. Lead exposure in children is linked to crime, violence, and juvenile delinquency [9, 10]. It has been reported that a significant contribution to the infant’s lead exposure is provided by the maternal’s lead transfer during pregnancy [11]. As a result, pregnancy is a critical time for exposure to lead from the mother to the developing foetus. Lead crosses the placenta freely [12] and in many cases, infants are already born with lead transferred to them from their mothers. Lead can adversely affect a range of birth outcomes, possibly by accumulating in the placenta and causing reduced nutrient transfer, oxidative stress, and abnormal function [13]. Poor birth outcomes are known to be associated with poor developmental trajectories in infancy, as well as having long-term implications for adult health [11]. There is an increased risk of kidney, cardiovascular and liver disease later in life due to lead toxicity [14]. Lead not only affects the developing foetus but also the pregnant woman [15]. During pregnancy, lead in maternal blood can direct to gestational hypertension [16], congenital malformations, spontaneous abortions, miscarriage, and stillbirths [15]. One of the adverse effects of lead exposure is pre-eclampsia, which is a silent killer and is associated with substantial maternal morbidity and mortality [17]. Therefore, lead exposure in pregnant women is a rising concern and requires an early risk assessment for better care planning.

Besides, the economic costs associated with childhood exposure to lead are substantial [18]. According to the World Bank analysis report 2020, the annual estimated economic costs of childhood lead exposure are at least $977 billion in low- and middle-income countries, $55 billion in the EU and $50.9 billion in the US [8]. On the other hand, the economic benefits of successful interventions against lead poisoning are substantial [19]. The benefits gained from successful lead exposure management far outweigh the costs of creating a national programme for screening, surveillance, and prevention of lead poisoning. In the current scenario, a practical approach to effectively determine lead toxicity levels is a lab-based blood test, which requires an expert medical/technical staff, expensive equipment like atomic absorption spectrometry and blood samples. This approach is inappropriate for lead screening in a large population as it is costly, time-consuming, infrequent, and resource exhaustive.

Even though relatively low levels of lead exposure may not adversely harm the mother but may influence the development of the foetus, and subsequent development and behaviour during childhood. Therefore, the effects of lead exposure need to be characterised to enable the delivery of an appropriate public healthcare system to lead-exposed women and newborn infants. It is significant to describe factors that contribute to maternal BLL, particularly those that could be reduced to minimise transfer to the developing foetus. In this context, multiple factors have been identified as probable contributors to maternal BLL. Maternal lead levels are high where there is direct exposure to environmental contamination, like areas of high pollution, living near lead-smelting or mining areas [3]. Apart from these exposures, there are other sociodemographic factors that directly/indirectly contribute to elevated BLL. Lead poisoning can occur through various sources like water, dust, soil, air, occupational, and take-home exposure [20–22]. Further, the use of cosmetics by women is a potential source of lead poisoning [23]. While research has demonstrated a link between lead poisoning in a pregnant woman and the developing foetus [11, 24], much of the sociodemographic factors influencing the cause remain unexplained. Quantifying sociodemographic factors and assessing early-stage lead toxicity levels in pregnant women could be beneficial in taking timely measures and reducing the effect of lead on the developing foetus.

Keeping the above view in mind, our research aims to explore sociodemographic features and machine learning (ML) techniques for maternal’s lead toxicity modelling within the context of developing countries. Using the aid of technology and through the integration of disciplines like Computing, Molecular Medicine, Biochemistry, Neonatology, and Gynaecology, the “Safe Motherhood Intervention” project is designed to deliver an affordable lead screening tool. The project aims to develop a low-cost point-of-care analytical tool such as a computational model in a form of a mobile-based application (app) that could predict lead toxicity levels in maternal blood without going for lab testing. The objectives of the project are described below:

1. Identify a set of sociodemographic features that could provide insight into lead toxicity levels in pregnant women,
2. Develop non-invasive and easy-to-access questionnaires based on the identified features,
3. Collect maternal data comprising umbilical cord blood and blood samples, and questionnaires reflecting lead exposure pathways,
4. Analyse the collected data and find the optimal set of features that support or do not support lead prediction modelling,
5. Estimate the underlying function and build the computational model framework, while keeping the size of the resulting model small and easy to interpret,
6. Evaluate the build framework performance in predicting the lead toxicity level based on the set of input features,
7. Design and develop the mobile-based screening app with the embedded prediction model.

The motivation of our work is directed towards gaining meaningful insight into the lead toxicity levels in pregnant women and including features that are more informative in modelling. This paper reports the first stage of the work and describes the initial set of results from the project. A set of sociodemographic features that indicate lead exposure pathway is identified and the questionnaires are developed. In the initial work, 200 subjects are recruited, and maternal data is collected, followed by feature selection, modelling and evaluation of the initial computational model framework built.

## Methodology

The work is a collaboration between Ulster University, UK, and the Indian universities: Era University, and King George Medical University.

### Questionnaire form

To identify relevant features that impact lead toxicity, interviews are performed with the various members of the research and development team, which included: biomedical engineers, computer scientists, neonatologists, and gynaecologists. Together, they identified a range of sociodemographic features potentially relevant to lead toxicity based on existing literature and their expertise. Information on environmental and lifestyle factors including data on age, cosmetic use, clinical history, highest educational qualification, housing type, fuel source, water source, smoking history, and exposure to passive smoking (partner or other household member smoking) are included in the questionnaire form.

**Fig. 1:**
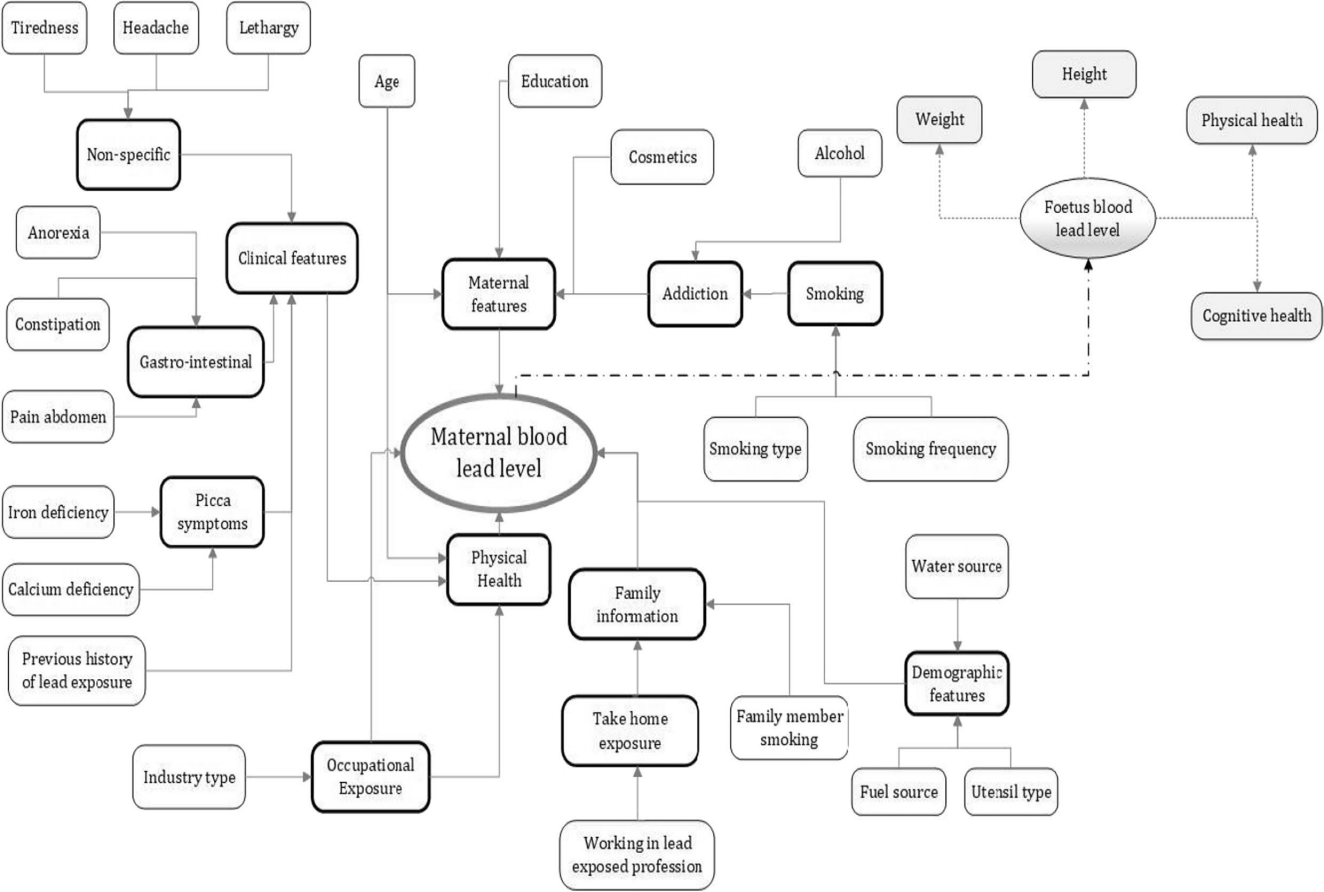
Influence diagram of features impacting maternal BLL and toxicity exposure

Features included in the questionnaire are significant and indicate typical exposure pathways of lead poisoning in pregnant women and consequently in the developing foetus. A feature like water sources is an important feature and signifies lead exposure pathways to the population at large that is not occupationally exposed. Water source and locality are the sources of baseline exposure to lead [22, 25, 26]. Lead exposure through smoking has a negative impact on pregnant women’s health and impacts the development and growth of the developing foetus [20]. Smoking is a common problem in India [20, 21] and including smoking-related questions is important. Therefore, in the questionnaire, two forms of smoking are included: (1) maternal smoking (type of smoking and number of times weekly smoking) and (2) family member smoking (type of smoking and number of times weekly smoking). A major part of lead toxicity results from its capacity to mimic other metals that take part in biological processes. Among the essential metals with which lead interacts are calcium, iron, and zinc [27]. Comorbidities like iron and calcium deficiency can enhance lead absorption. Anaemia is a common manifestation of lead toxicity and iron deficiency often coexists with lead intoxication [28]. The prevalence and severity of lead-induced anaemia relate directly to the blood lead concentration. Younger and iron-deficient children are at greater risk of lead-induced clinical anaemia [29]. Calcium and iron intakes appear to be inversely associated with blood lead concentrations in pregnancy [11]. Therefore, iron and calcium deficiencies are included in the questionnaire. Figure 1 shows an influence diagram indicating the set of features that have an association with maternal’s BLL and affect lead toxicity exposure.

The influence diagram illustrates the relationships between the features themselves and more significantly, the relationship with the maternal’s BLL. The diagram comprises independent features (enclosed within the rectangles with thin lines) and summary features (enclosed within the rectangles with thick lines). Though summary features are influenced by independent features, they may also be a feature in their own right. For example, in Fig. 1, the independent features, age and education are likely to influence industry type (a summary feature), which consecutively can influence occupational exposure (a summary feature) and subsequently may affect a maternal’s BLL. Figure 1 additionally shows the perceived effect of lead toxicity on the developing foetus. It is perceived that the foetus’s BLL is impacted by the mother’s BLL and affects the baby’s weight, height, and physical and cognitive health.

### Data collection

Following the identification of potential features pertaining to maternal lead toxicity levels, the study is designed to collect maternal data comprising sociodemographic features and blood samples. Before the start of the study, prior approvals are taken from the institutional review board and the data is collected over a period of 3 months.

#### Inclusion and exclusion criteria

The study included only those subjects who are above 18 years of age and have given informed written consent to take part in the study. The study excluded those subjects who are below 18 years of age and/or refused to give consent for the study.

#### Subject recruitment

The study is carried out at Era's Lucknow Medical College and Hospital. Pregnant women visiting the hospital for delivery are explained the significance, need, and design of the study. Informed, written consent is taken from the subjects eligible to participate in the study. Given the nature of the study along with a limited time and budget, the study enrolled 200 pregnant women, and the umbilical cord blood and blood samples are taken i.e., a total of 400 blood samples. Prior to the use of data, the data is anonymised to remove all sensitive information.

#### BLL estimation

The BLL estimation is carried out by collecting 2 mL of venous blood of the mother and 2 mL of umbilical cord blood in ethylene diamine tetra-acetic acid (EDTA) vacutainers. The samples are labelled and kept in a cooling box unless analysed. The collected mother and umbilical cord blood samples are analysed with the help of an inductively coupled plasma-optical emission spectrophotometer (ICP-OES), model Optima 8000, Perkin Elmer, USA) after prior microwave digestion. Table 1 details the experimental setup followed for the BLL determination.

**Table 1:** Experimental set-up for the BLL determination

Reagents	Nitric acid, perchloric acid, hydrogen peroxide, Milli-Q water	
Glassware	Graduated pipette, test tube and vacuum dispenser	
Metals standard solution	Stock solutions of studied elements at 1000 mg/L concentration were obtained. Working the standard for LEAD analysis is prepared by diluting stock solution in 2% nitric acid in the desired range	
Sample	0.5 mL to 2.0 mL whole blood in EDTA vial	
Operating conditions for ICP-OES	Plasma gas flow (L/min)	8
	Auxiliary gas flow (L/min)	0.2
	Carrier gas flow (L/min)	0.55
	RF power (W)	1300
	View distance	15
	Plasma view	Axial
	Sample flow rate (mL/min)	1.0
	Wavelength (nm)	220.353

a. *Microwave digestion* we used the microwave digestion technique as described in [30] with a little modification. The blood samples are digested with the help of a Microwave Reaction system (Multiwave 3000, Anton Paar, Perkin Elmer) with the Rotor 16HF100 (100 mL PFA vessels, 40 bar) and pressure–temperature (p/T) sensor. Blood samples (0.5 mL) are digested with 2.0 mL of HNO_3_:HCIO_4_ (3:1), 1.0 mL of H_2_O_2_ and 2.0 mL of H_2_O_2_ in the microwave digestion system. The microwave reaction system is programmed to attain 400 W of power with 5 min ramping hold for 10 min and then increased to power 800 W with 5 min ramping to hold for 10 min, then cooled to 0 power. A blank using Milli-Q water in place of the sample is also digested and prepared. The resulting clear solution after microwave digestion is cooled and analysed for lead levels.

b. *ICP-OES analysis* the resulting clear solutions obtained after microwave digestion are analysed by ICP-OES for lead level using the Certified Reference Material (CRM) provided by Perkin Elmer, USA [31]. The ICP-OES operating conditions are described in Table 1. The ICP-OES instrument (Perkin Elmer Optima 8000, USA) is calibrated with different concentrations of lead standard. The working calibration standard solutions range from 0.005 to 1.0 mg/L of lead (Pb) and are prepared from a stock standard of 1000 mg/L by dilution in 0.2% nitric acid. A calibration curve is prepared with a correlation coefficient of 0.9999 using linear through zero (Fig. 2). The calibration blank is also prepared using Milli-Q water instead of the Pb standard. The samples and blank solutions after microwave digestion are analysed and the results of BLL are expressed in µg/dL. The recovery and limit of detection of lead are also carried out by spiking the blood samples with different concentrations of lead standard. More than 90% of recoveries are obtained with a limit of detection of 0.001 mg/L.

**Fig. 2:**
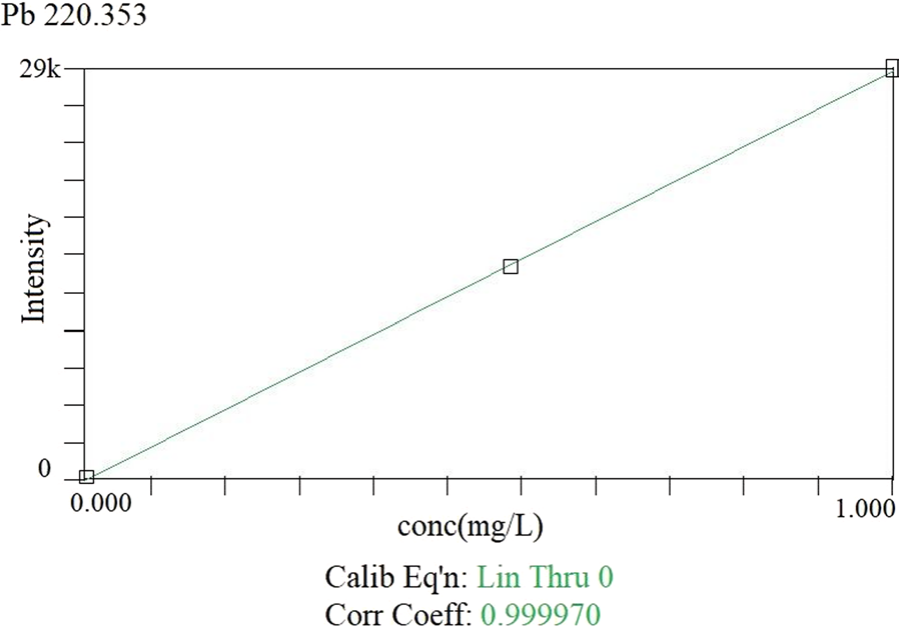
Lead calibration graph

### Data analysis

The determined BLL values and details collected from the questionnaire form are collated together to make the maternal dataset of 200 samples. In total 27 features are collected along with gestation age, baby weight (kg), and BLL values of the mother and the newborn (Table 2).

**Table 2:** List of features collected in the dataset

Feature details		
Age (years)	Cosmetic use	Occupational exposure
Education	Water source	Take-home exposure
Mother smokes (yes/no)	Family member smoking per week	Non-specific
Mother smoking type	Family member alcohol	Gastro-intestinal
Mother smoking per week	Family member alcohol times/week	Pica symptoms
Alcohol (yes/no)	House—how much old	Previous history of lead exposure
Alcohol per week	Recent paint	Utensils
Smoking family member (yes/no)	Recent plumbing	BLL in mother (μg/dL)
Family member smoking type	Distance from traffic congestion (km)	BLL in child (μg/dL)
Fuel source	Living near industry	

The collated data is pre-processed to clean incorrect, incomplete, inconsistent, improperly formatted, duplicate, and missing values. Consequently, the data dictionary is created to convert the data from raw format to another format to allow for more convenient consumption of the data. For example, ‘cosmetic use’ is a single feature having collective information for the use of dye, kohl, lipstick, sindoor, and surma. However, each of these cosmetics has a different level of impact on BLL, therefore they are considered separate features in the analysis. In the collected dataset, each feature has many categories which means the built models are relatively complex with a high probability of lower accuracy [32]. For instance, age is a continuous value and education has too many categories. Since these features typically have too many categories, statistical tests like the Chi-squared test may have few instances of data in some categories, and this can weaken the test [32]. We, therefore, categorise each feature into fewer values based on relevance. Initial feature reduction is carried out and a manual screening is undertaken to prune out irrelevant features. The 27-feature set is consequently reduced to an 18-feature set. Table 3 provides details of the 18-feature set labelled with fewer categories.

**Table 3:** Details of the 18-feature set labelled with fewer categories

Personal	Personal features of a mother	
	Age	gretaerThan30, lessThanEqual30
	Education	College_HigherDegree, NoCollege
	Mother smoking	Yes, no
	Occupational exposure	Housewife, office
	Dye	Yes, no
	Kohl	Yes, no
	Lipstick	Yes, no
	Sindoor	Yes, no
	Surma	Yes, no
Second-hand exposure	Features that account for maternal lead level due to the smoking of any family members	
	Family member smoking	Yes, no
	Family member smoking type	Smoking type
Clinical	Clinical manifestation of lead and describe the health conditions of an individual	
	Nonspecific	Headache, Headache_Lethargy, Headache_Tiredness, Lethargy, Lethargy_Tiredness, No, Tiredness
	Gastrointestinal	Anorexia, Anorexia_PainAbdomen, Constipation, No, PainAbdomen
	Pica symptoms	CalciumDeficiency, IronDeficiency, No
Household	Water source	GroundWater, GroundWater_ ROWater, ROWater, GroundWater_TapWater, ROWater_TapWater, TapWater
	Utensils	Aluminium, Aluminium_Ceramic_Steel, Aluminium_Steel, Other_Steel, Steel
	Fuel source	Kerosene, LPG, Wood
Take-home exposure	Lead exposure due to family member/s working in lead-based industries	
	Agriculture, AutoDriver, AutoDriver_Ceramics, AutoRepair, Batteries, Batteries_Lock, Ceramics, Construction, Construction_Furniture, Construction_Painting_Plastic_Polishing, Furniture, None, Other, Painting, Painting_Furniture, Painting_Polishing, PipeFitting, PlasticManufacturing, PlasticManufacturing_Soldering, Polishing, Polishing_Soldering, Soldering, Steel	

**Fig. 3:**
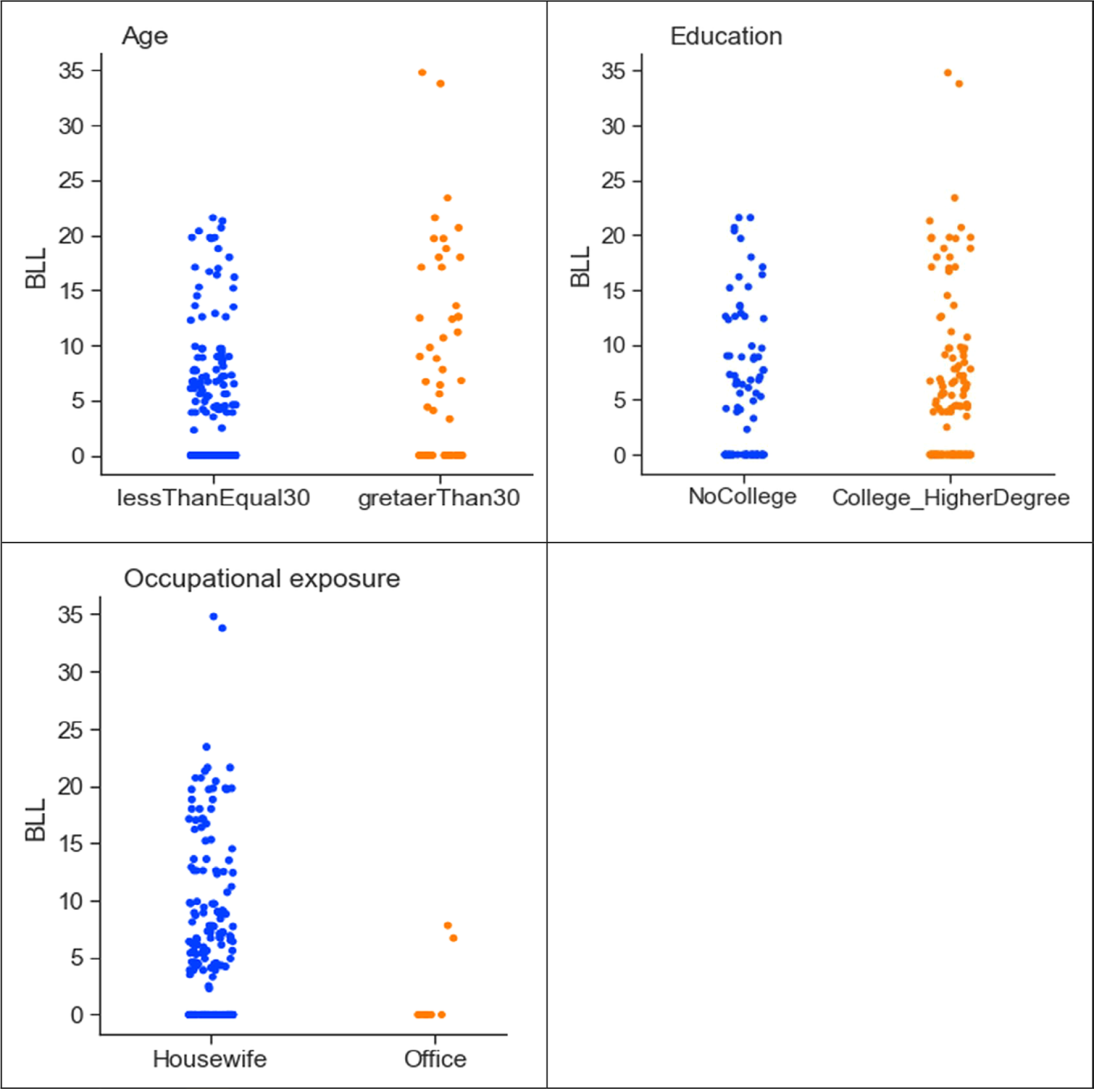
Correlation of lead concentration with mother’s age, education, and occupation

The maternals’ BLL values ranged from not detected (ND) to 35 μg/dL. The age feature is characterised as less than or equal to 30 and greater than 30 (Table 3). The education feature is categorised as “no college” (includes uneducated and education up to class 12th) and “college and higher degree” (includes graduate and higher degree). Occupational exposure indicates direct job working exposure to lead. The mother’s occupation is categorised into two types “housewife” and “office”, based on the collected data. To find the correlation between the BLL and the identified features, descriptive analysis is carried out in Python. The correlation between a mother’s age, education, and occupation with lead concentration is shown in Fig. 3.

**Fig. 4:**
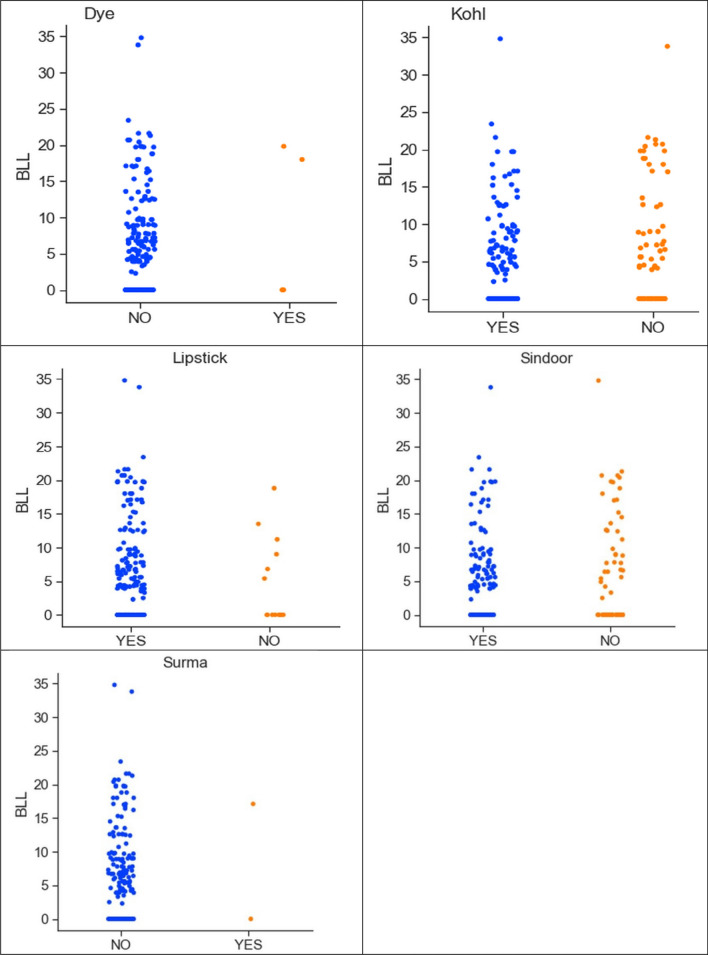
Correlation between lead concentration and use of cosmetics

**Fig. 5:**
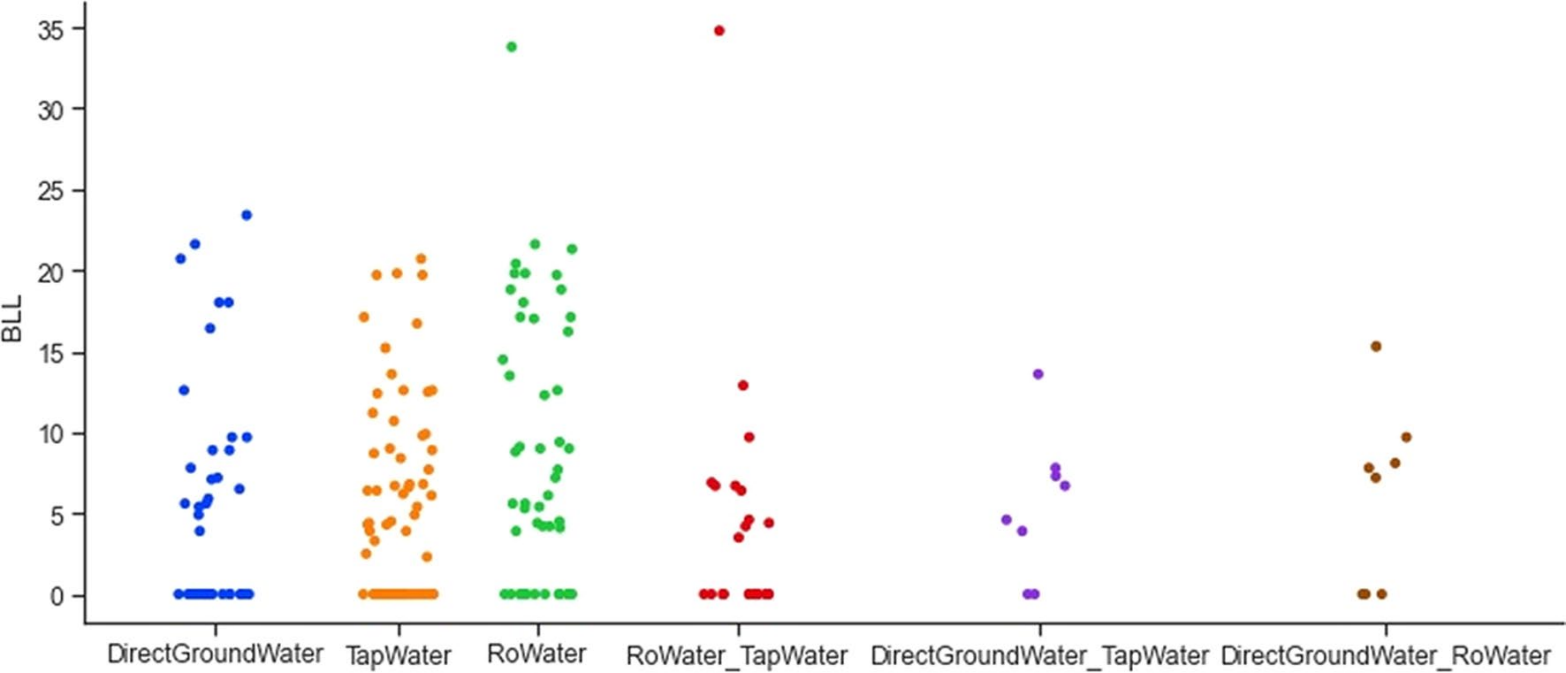
Correlation between different water sources and lead concentration

The collected data suggests a slightly higher level of lead concentration in pregnant women who are aged above 30. Figure 4 shows the correlation between lead concentration and the use of dye, kohl, lipstick, sindoor, and surma. From Fig. 4, it can be observed that the mothers who used kohl, lipstick, and sindoor have elevated levels of lead in the blood samples. This confirms the fact that lead-based cosmetics can be the reason for the elevated BLLs. The collected dataset has information for different sources of water: groundwater sources (tube well and submersible), tap water through pipelines, reverse osmosis (RO) water, use of both tap water and RO water, use of both groundwater and RO water, and use of both tap water and groundwater (Table 3). Figure 5 shows the correlation between different water sources and lead concentration.

**Fig. 6:**
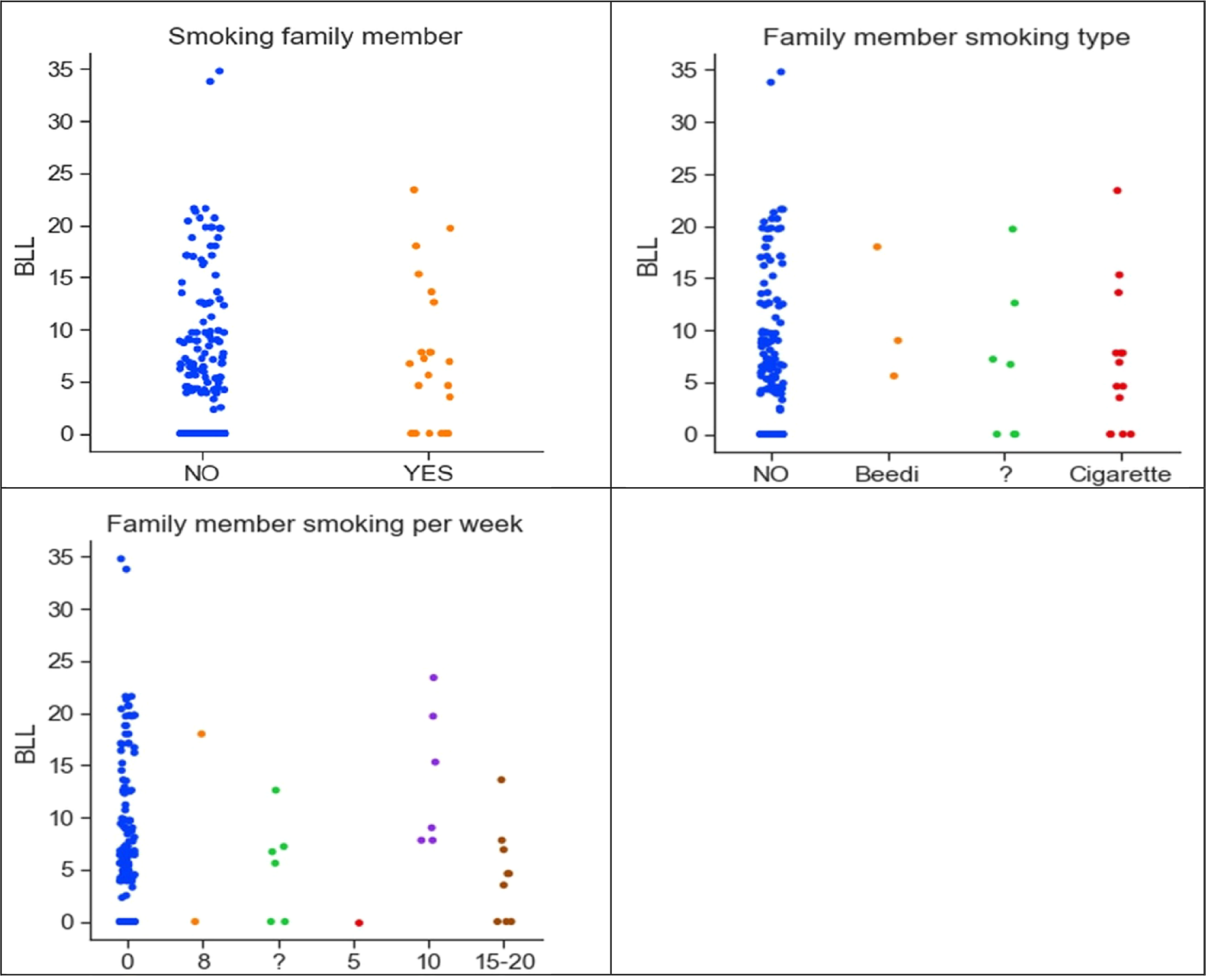
Correlation between lead concentration with family member smoking, type of smoking, and the number of times they smoked per week

Second-hand smoking exposure due to smokers in the family is also a reason for a higher level of lead concentration in pregnant women and therefore, this information is also included in the dataset. Two features included in the study are the mother smoking and family member smoking. In the collected dataset, only one mother has a smoking habit. Figure 6 shows a correlation between lead concentration and family member smoking, type of smoking, and the number of times they smoked per week.

**Fig. 7:**
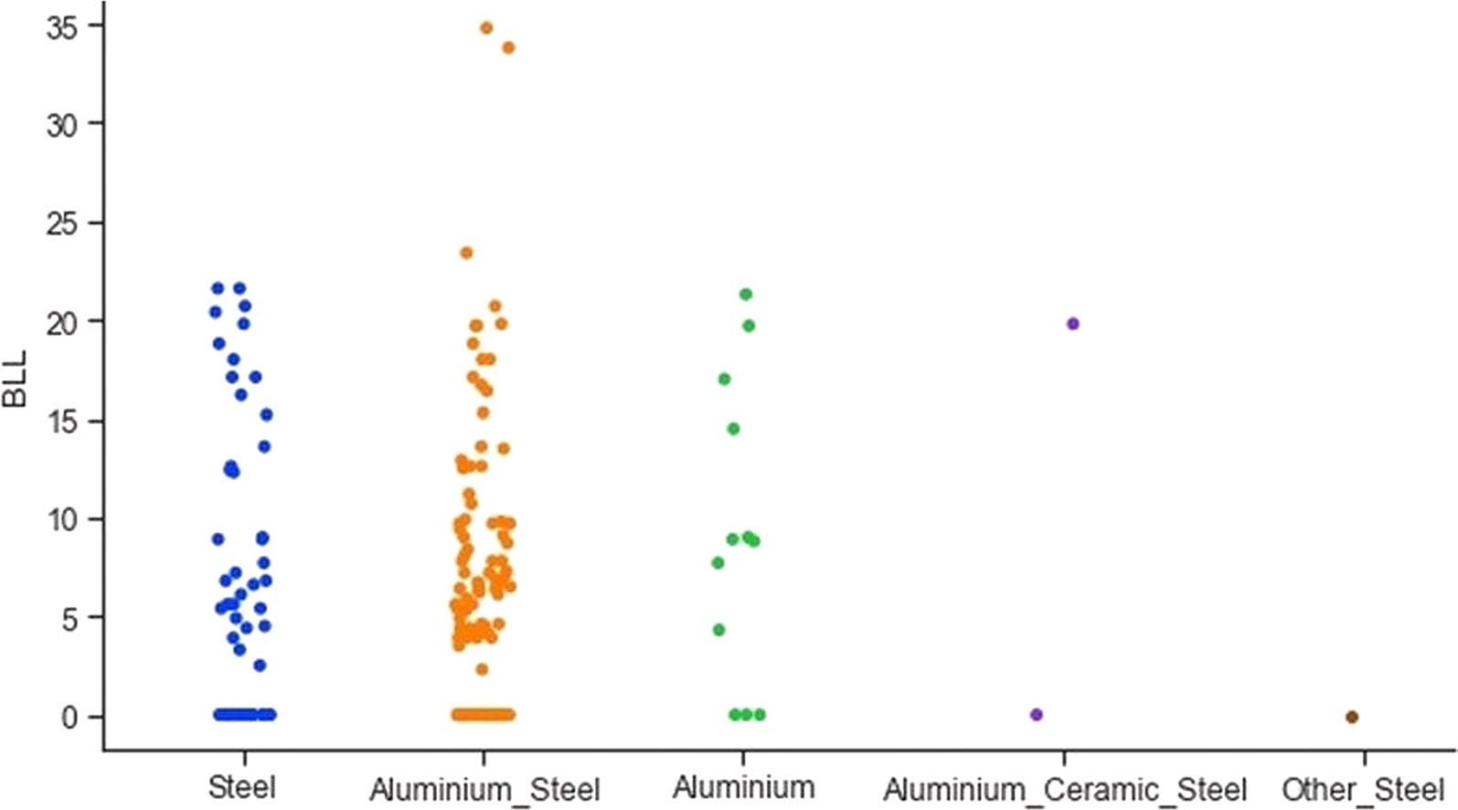
Correlation between different types of utensils and lead concentration

**Fig. 8:**
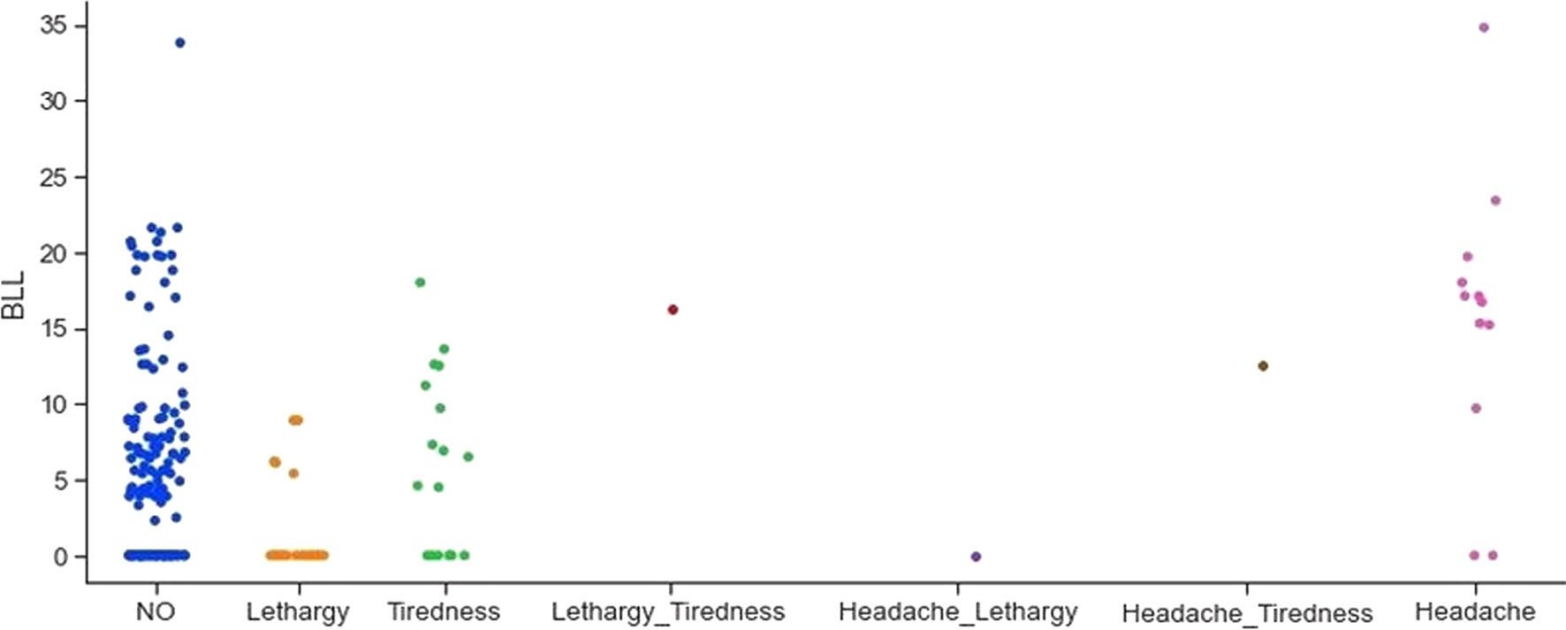
Correlation between non-specific generalized symptoms and lead concentration

The next feature analysed is the type of utensils used for cooking. The utensil feature is categorised as steel, aluminium, both steel and aluminium, others and steel, and aluminium, ceramic, and steel. Figure 7 shows the correlation between different types of utensils and lead concentration. From Fig. 7, it can be observed that mothers using aluminium and steel do have elevated BLL values. In the collected dataset, information for non-specific generalised symptoms, related to lead toxicity, like lethargy, tiredness, and headache are also included. Figure 8 shows that mothers having these non-specific generalised symptoms have a higher lead concentration in the blood.

**Fig. 9:**
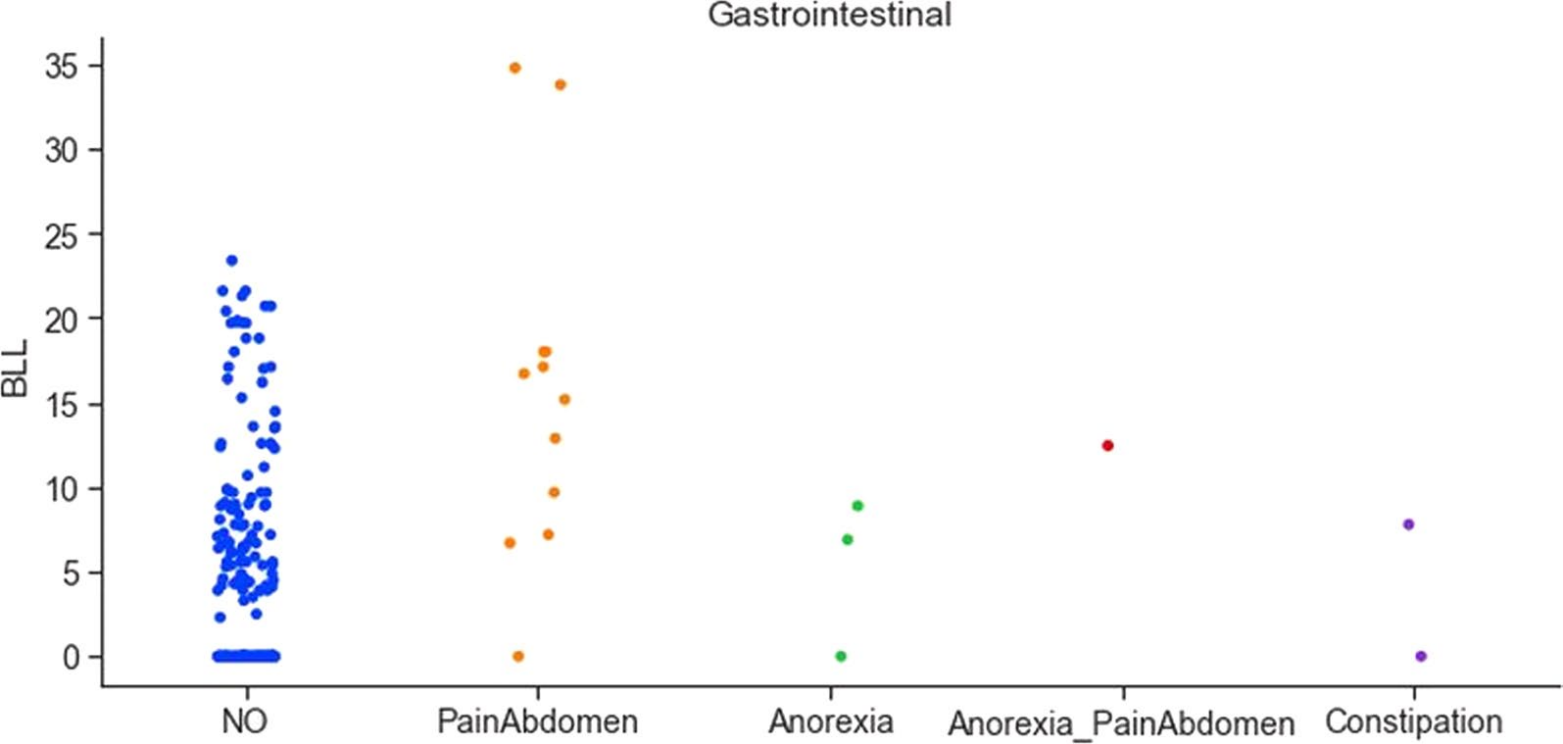
Correlation between pica symptoms and lead concentration

**Fig. 10:**
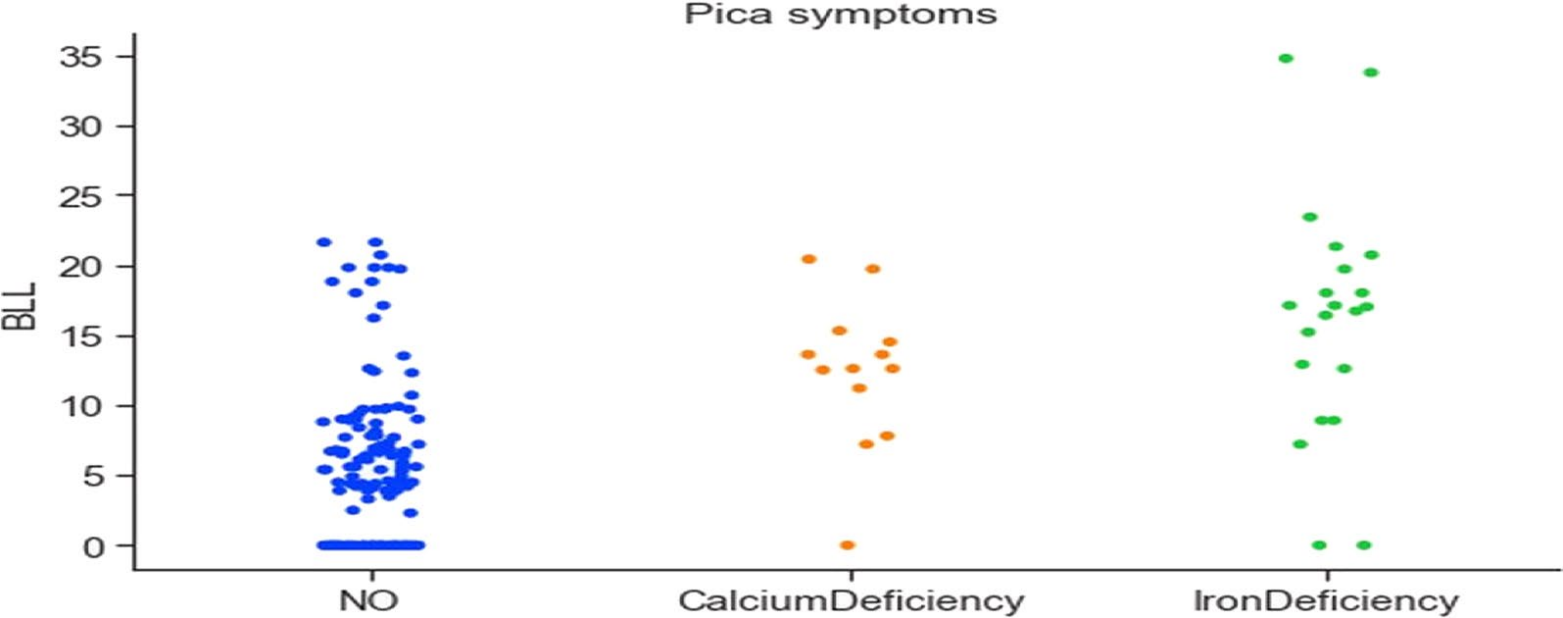
Correlation between pica symptoms and lead concentration

Gastrointestinal manifestations of lead poisoning include chronic or recurrent abdominal pain [33]. Figure 9 shows a high correlation between abdomen pain and elevated BLLs. The data also reported pica symptoms in individuals. Figure 10 shows the correlation between pica symptoms and lead concentration. The prevalence and severity of lead-induced anaemia relate directly to the blood lead concentration. The collected data confirms this fact, Fig. 10 shows a high correlation between iron deficiency and lead toxicity. Also, in the literature, calcium deficiency has been linked to increased lead absorption [34]. The collected dataset indicates that individuals having calcium deficiency had higher lead concentrations (Fig. 10).

**Fig. 11:**
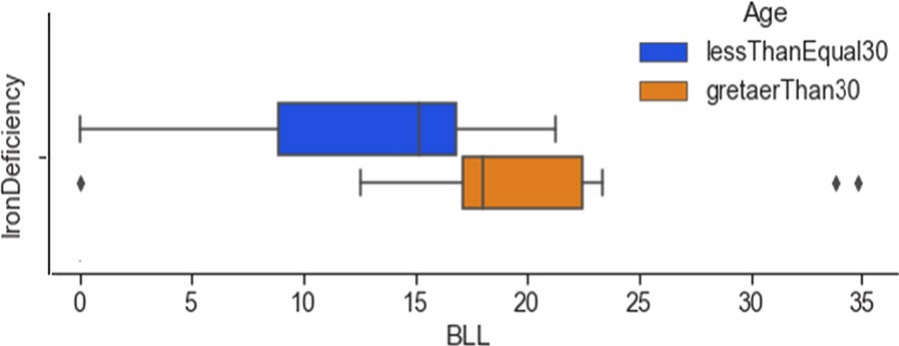
Box plot for iron deficiency versus age with respect to lead concentration

The existing literature reports that long-time exposure to lead can be the cause of anaemia among middle and older-aged people [35]. It would be interesting to see if the collected data reflects this pattern. Figure 11 shows the box plot of age versus iron deficiency with respect to lead concentration. Subjects who were above 30 and had an iron deficiency also had elevated lead concentration, reflecting a long time of exposure to lead.

Women can be exposed to lead even by handling or washing their family’s lead-contaminated clothes [36]. Some of the jobs do come with the potential of more obvious lead exposure. For example, battery manufacturing/repair, construction, lead smelting, soldering, plumbing, auto-body repair, pottery, rubber and plastics manufacturing, stained glass, tile, ceramics, and manufacturing or using leaded paints, dyes, glazes, inks, or pigments. The collected dataset studied the occupation of family members for potential risk of take-home exposure and the details are provided in Table 4. The analysis reiterated the association between lead exposure and certain jobs. Pregnant women whose family members worked in plastic manufacturing, polishing, auto driving, soldering, pipefitting, battery manufacturing and repairing, construction, auto repair, and painting had higher lead concentrations. It is to be noted that certain combinations of jobs reflected very high lead exposure in a pregnant woman. This includes Polishing_Soldering (*mean BLL* = 19.7 μg/dL), Painting_Furniture (*mean BLL* = 11.05 μg/dL), and Construction_Painting_Plastic_Polishing (*mean BLL* = 12.6 μg/dL). For fuel sources and alcohol use, there is very limited data in each category, so it has not been shown in separate figures.

**Table 4:** Summary of the findings from the analysis of the data

Sociodemographic characteristics		Sample size (*N*)	BLL mean (μg/dL)
Age	lessThanEqual30	150	5.45
	gretaerThan30	50	8.42
Education	NoCollege,	82	6.07
	College_HigherDegree	118	6.28
Mother smoking	Yes	1	0
	No	199	6.23
Occupational exposure	Housewife	191	6.41
	Office	9	1.61
Pica symptoms	No	165	4.47
	IronDeficiency	21	16.18
	CalciumDeficiency	13	12.38
	Unknown	1	0.00
Family member smoking type	Cigarette	14	6.81
	Beedi	3	10.87
	Unknown (?)	7	6.60
	No	176	6.05
Water source	TapWater	68	5.48
	ReverseOsmosisWater	51	8.74
	DirectGroundWater	41	5.57
	ReverseOsmosisWater_TapWater	24	4.20
	DirectGroundWater_TapWater	8	5.49
	DirectGroundWater_ ReverseOsmosisWater	8	6.01
Non-specific	No	149	5.90
	Lethargy	19	1.87
	Tiredness	17	6.32
	Headache	12	15.58
	Lethargy_Tiredness	1	16.20
	Headache_Tiredness	1	12.60
	Headache_Lethargy	1	0.00
Dye	Yes	4	9.45
	No	196	6.13
Kohl	Yes	132	5.58
	No	68	7.39
Lipstick	Yes	186	6.31
	No	14	4.62
Sindoor	Yes	125	6.35
	No	75	5.92
Surma	Yes	2	8.55
	No	198	6.17
Smoking family member	Yes	23	7.18
	No	177	6.07
Gastrointestinal	No	182	5.56
	PainAbdomen	12	15.84
	Anorexia	3	5.27
	Constipation	2	3.9
	Anorexia_PainAbdomen	1	12.6
Utensils	Aluminium_Steel	129	5.62
	Steel	55	6.74
	Aluminium	12	9.27
	Aluminium_Ceramic_Steel	2	9.90
	Unknown	1	12.60
	Other_Steel	1	0.00
Fuel source	LPG	196	6.12
	Kerosene	2	10.8
	Wood	2	8.55
Take-home exposure	None	104	4.94
	AutoDriver_Ceramics	1	0.00
	Batteries_Lock	1	6.10
	Construction_Furniture	1	6.80
	PlasticManufacturing_Soldering	1	0.00
	PlasticManufacturing	1	34.80
	Steel	1	0.00
	Painting_Polishing	1	4.40
	Polishing	1	0.00
	Furniture	11	5.28
	Painting	10	8.69
	Soldering	1	9.40
	AutoRepair	9	10.35
	Painting_Furniture	8	11.05
	Ceramics	5	3.42
	Batteries	4	15.90
	Agriculture	2	2.45
	PipeFitting	1	16.40
	Construction_Painting_Plastic_Polishing	1	12.60
	Polishing_Soldering	1	19.7
	AutoDriver	1	8.90
	Other	21	4.13
	Construction	13	8.28

Table 4 also details the summary of the findings from the analysis of the data. From the analysis, it can be concluded that certain features are significant and contribute to elevated lead levels in pregnant women. Features like active and passive smoking are important, especially in the context of developing countries like India, where tobacco exposure is a major concern. The analysis found that women who were exposed to second-hand smoke had elevated BLLs (Overall mean BLL = 7.18 μg/dL, beedi mean BLL = 10.87 μg/dL, cigarettes mean BLL = 6.81 μg/dL). Subjects who were aged above 30 had a higher lead concentration (mean BLL = 8.42 μg/dL), indicating that long-time exposure to lead has built up in the body. Lead is cumulative and the use of cosmetic products can bring about potential exposure to its toxicity. In the collected data, it was found that the subjects using cosmetics such as dye (mean BLL = 9.45 μg/dL), kohl (mean BLL = 5.58 μg/dL), lipstick (mean BLL = 6.31), sindoor (mean BLL = 6.35 μg/dL), and surma (mean BLL = 8.55 μg/dL) had elevated BLLs. The use of RO filters removes the lead from water, however in the collected dataset, surprisingly the subjects who used RO water had higher BLLs (mean BLL = 8.74 μg/dL). This may be due to other features causing elevated BLLs. It was also found that the use of aluminium-based utensils can be the reason for higher lead concentrations (mean BLL = 9.27 μg/dL). The clinician manifestation of lead toxicity is reflected in the non-specific, pica symptoms, and gastrointestinal features. The existence of headaches among 12 subjects was highly correlated to elevated lead concentration (mean BLL = 16.20 μg/dL). Also, pain in the abdomen was highly correlated to the elevated lead concentration (mean BLL = 12.60 μg/dL). The analysis found very high lead levels in 21 subjects who had iron deficiency (mean BLL = 16.18 μg/dL) and in 13 subjects who had calcium deficiency (mean BLL = 12.38 μg/dL). The take-home lead exposure due to family member/s working in lead-based professions was high, specifically in jobs like construction (mean BLL = 8.28 μg/dL, 13 subjects), painting (mean BLL = 8.69 μg/dL, 10 subjects), auto repair (mean BLL = 10.35 μg/dL, 9 subjects), painting and furniture (mean BLL = 11.05 μg/dL, 8 subjects), and batteries (mean BLL = 15.90 μg/dL, 4 subjects). The lead levels were also high in professions such as soldering, polishing, plastic manufacturing, pipe fitting, and auto driving. However, we have a limited number of subjects in these categories to draw a conclusion regarding the association between lead toxicity and these professions.

**Fig. 12:**
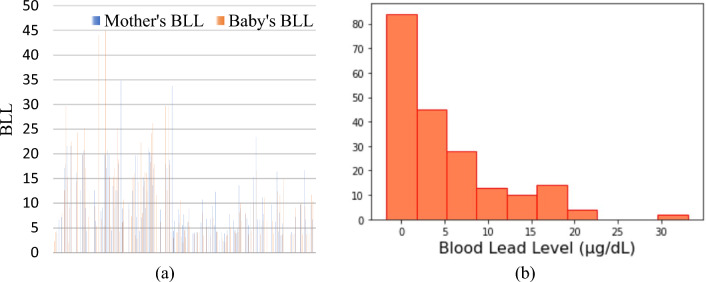
**a** Relative plot of mother’s and baby’s BLL values. **b** Histogram of mother’s BLL values

Figure 12a shows a relative plot of the mother’s and baby’s BLL values. The plot indicates that babies, whose mothers had relatively higher BLL values, had higher BLL values in most of the cases. This confirms that a significant amount of lead transfers from the mother to the foetus.

### Predicting lead toxicity level

The focus of our work is to analyse the pregnant women’s background for the potential risk of lead toxicity. The collected maternal data is used to build a computational model that takes in the sociodemographic features and predicts the lead toxicity level. It is anticipated that such a computational model can analyse personal, second-hand smoking, take-home exposure, general, and clinical features and then can predict the output class variable, *lead content level*.

The range of BLL values that should be considered as high toxicity levels vary from country to country and the current permissible BLL limit is set to 10 μg/dL by the WHO. However, the BLL value of 10 μg/dL, previously measured to be safe is now considered unsafe for health and harms multiple organs, even in the absence of explicit symptoms [37]. However, recent studies have found that neuro-behavioural damage occurs at a BLL of 5 μg/dL and even lower. Therefore, a definite BLL threshold below which lead causes no injury to the developing brain is difficult to identify. Lately, the Centres for Disease Control and Prevention have set a new threshold of 5 μg/dL concerning childhood BLLs [38]. In the collected dataset of 200 samples, the BLL values ranged from 2.3 to 34.8 µg/dL. The sample also contains no ND values. These are recorded for those cases where lead is not detected in the blood sample. There are 81 data points with ND as a BLL value. The BLL values are discretised into discrete states to incorporate them into the model. It is postulated that directly using continuous lead values may result in making the model sensitive to variations in the values [39]. Nevertheless, directly incorporating continuous lead values (the measured BLL) into a model is a complex and computationally intensive process that may not prove to be very informative for prediction [39]. Also, a large amount of data is required for such an approach, therefore we focused on using discretised BLL values. Because the primary focus of this paper is to exhibit the use of sociodemographic features in building a prediction model that can predict *lead content level*, we here follow a simple process of visual inspection to discretise the BLL values. However, there are other methods to discretise the continuous data, which are detailed in [39]. Figure 12b shows a histogram of BLL values and their frequency in the collected dataset. There is a clear separation between the data, resulting in clear distinct clusters; thus, making the visualisation straightforward. Based on this representation, the data is therefore discretised into five clusters and the output class variable, the *lead content level* is labelled as ND, LessThan5, Between5_10, Between10_15, and GreaterThan15.

### Classification algorithms

For developing the most suitable algorithm for predicting the *lead content level*, a range of data mining algorithms are explored to find their suitability in building the prediction model [40]. Table 5 presents the details of the algorithms and parameter settings used in our case.

**Table 5:** Classification algorithms details and parameter setting used in building prediction models

Classification algorithms	Details	Parameter settings
*Neural Network* (NN)	Consists of artificial neurons that are connected to each other following a specific architecture. NN transforms the inputs into meaningful outputs	Number of hidden layers *a* = (#attributes + #classes)/2 Learning rate = 0.3
*C4.5 Decision Tree* (*DT*)	A DT algorithm is based on divide and conquer. The splitting criterion is based on the feature with the highest normalized information gain, where information gain is calculated using entropy between the nodes prior to and post-splitting the feature	Un-pruned; minimum number of leaf nodes = 1
*Support Vector Machine* (SVM)	An SVM classifier is trained using Platt’s sequential minimal optimisation algorithm. SVM uses hyper-planes to distinguish and maximises the margin between data of different classes	Complexity parameter *c* = 1.0; Poly-Kernel
*Adaptive Boosting* (AB)	AB is an ensemble learning algorithm, which uses multiple learners to solve a problem. The final model is derived by weighted majority voting of the base learners from weighted training data where the weights are updated in the previous test process	Number of iterations = 10; base classifier: decision stump
*k-Nearest Neighbour* (*k*NN)	*k*NN classifiers find a group of *k* objects in the training set that is closest to the test object and the assignment of the label is based on the predominance of a particular class in this neighbourhood	*k* = 1; nearest neighbour search algorithm; linear search
*Classification and Regression Trees* (CART)	The CART DT is a binary recursive partitioning procedure capable of processing continuous and nominal attributes both as targets and predictors. Data are handled in their raw form	Heuristic search
*Naïve Bayes* (NB)	NB is a probabilistic classifier based on Bayes’ theorem with strong (naive) independence assumptions that assumes the presence of a particular feature of a class is unrelated to the existence of any other feature, given the class variable	–

### Handling imbalanced classes

In the collected dataset, the output class variable, *lead content level*, has a varying number of data points for each of the categories: ND = 81, LessThan5 = 24, Between5_10 = 52, Between10_15 = 14) and GreaterThan15 = 29. As a result, the given data is imbalanced and this imbalance in the class sizes can influence the outcome of some of the classification algorithms, typically with a bias towards the majority class (i.e., the one which has a higher number of values) [32]. The size distribution of the classes is 40.5% ND, 12% LessThan5, 26% Between5_10, 7% Between10_15, and 14.5% GreaterThan15. Due to this imbalance in the collected data, the fitted models have a higher possibility of incorrectly classifying most of the unknown instances to the majority class, i.e., the ND class in our case. Therefore, in this work, we also investigated the benefits gained by using resampling techniques.

The objective of undertaking resampling is not to improve the accuracy but to handle the bias. If the classes are not balanced, the majority class will dominate and in extreme cases all the unknown cases get assigned to the majority class, thus leading to misclassification [32, 41]. The purpose of the resampling technique is to let the model classify the unknown cases solely based on the robustness of a classification algorithm and the merits of the selected features. Therefore, to build more accurate prediction models and minimise bias, the imbalance in the data is handled by applying a resampling technique.

To handle the imbalance between the classes, the Synthetic Minority Over-Sampling Technique (SMOTE) is applied to the data. SMOTE is a commonly used technique due to its effectiveness and simplicity [42]. The given data is resampled using the SMOTE filter in WEKA Experimenter (University of Waikato, Version 3.8) and the classes are given a boost as shown in Table 6. In Weka, the new instances of the minority class created are added at the end of the data. Therefore, the order of appearance of the data instances is subsequently randomised to avoid overfitting. This is performed using the Randomize filter in the Weka Experimenter.

**Table 6:** SMOTE applied to the four minority classes

Majority class: ND (*n* = 81)	
Minority class	Percentage boost (%)
LessThan5 (*n* = 24)	(81 − 24)/24 * 100 = 237.5
Between5_10 (*n* = 52)	(81 − 52)/52 * 100 = 55.77
Between10_15 (*n* = 14)	(81 − 14)/14 * 100 = 478.57
GreaterThan15 (*n* = 29)	(81 − 29)/29 * 100 = 179.31

Following the process of resampling, the new data is an 18-feature dataset with 81 instances each of ND, LessThan5, Between5_10, Between10_15, and GreaterThan15 each and in a total of 405 instances. In the given data, the ND values are assigned to those instances, where the BLL is less than or equal to 3 μg/dL. However, the ND and LessThan5 values are two clusters very close to each other with respect to the BLL values and therefore, can be merged into one cluster. Consequently, in the next modelling phase, we merged ND and LessThan5 labels, resulting in a new class label, ND_5. The new output classes are ND_5 (105), Between5_10 (52), Between10_15 (14), and GreaterThan15 (29), which is still imbalanced data. Therefore, again the SMOTE is applied to rebalance the data based on the output classes ratio, followed by the randomisation technique.

### Feature selection

In the next step, feature selection is carried out to identify features that can make a good prediction of class membership for the output classes under investigation.

### Univariate analysis

A univariate analysis is carried out by applying the Chi-square test on each of the 18 features against the output class, *lead content level*. While applying the pair-wise test, those cases which have the missing information are dropped as these cases do not add up to any information in finding a relationship between the two variables (i.e., the feature and the output class variable) [32, 41]. A Chi-square test is applied to the 18-feature set with four class labels (ND_5, Between5_10, Between10_15, and GreaterThan15). Based on the *p*-values obtained for the features, Age (*p*-value = 0.008), TakeHomeExposure (*p*-value = 0.019), NonSpecific (*p*-value = 0.000), GastroIntestinal (*p*-value = 0.000), and PiccaSymptoms (*p*-value = 0.000) are found to be significantly associated with the output class, *lead content level*.

### Multivariate analysis

In the influence diagram, shown in Fig. 1, a few of the features are directly related to the output class, whereas a few of them may be indirectly related. The univariate analysis showed a limited direct correlation between the features and the output classes. This may be because the Chi-squared test may not be able to identify all the relevant features. The multivariate analysis produces more accurate feature ranking by evaluating multivariate statistics that consider the dependencies among features when calculating feature scores. Keeping this view in mind, next the multivariate analysis is carried out to find which combination of features can work best to predict the *lead content level*, reduce the computational complexity, increase the accuracy of the built models, and optimise the cost of feature selection. Here we apply two multivariate feature selection methods namely, the multinomial logistic regression and the Boruta algorithm.

### Multinomial logistic regression

Logistic regression is a technique used when the dependent variable is categorical (i.e., nominal). For binary logistic regression, the number of dependent variables is two, whereas the number of dependent variables for multinomial logistic regression is more than two. Multinomial logistic regression is an extension of binary logistic regression that allows for more than two categories of the dependent or outcome variable [43]. Table 7 shows the likelihood ratio tests obtained by applying the multinomial logistic regression to the data with four class labels. From the analysis, out of 18 features, 8 features are found to be significant and have a *p*-value < 0.05.

**Table 7:** Likelihood ratio tests table obtained by applying multinomial logistic regression

Effect	Model fitting criteria	Likelihood ratio tests		
	− 2 Log likelihood of reduced model	Chi-square	df	Sig
Intercept	293.767^a^	0.000	0	
Age	297.051^b^	3.284	3	0.350
Educ_Categorised	304.965^b^	11.198	3	**0.011**
MotherSmoking	293.767^a^	0.000	0	
SmokingFamilyMember	294.359^b^	0.592	3	0.898
FamilyMemberSmokingType	306.718^b^	12.951	9	0.165
OccupationalExposure	305.528^b^	11.761	3	**0.008**
TakeHomeExposure	472.219^b^	178.452	63	**0.000**
WaterSource	358.297^b^	64.530	15	**0.000**
Dye	294.150^b^	0.383	3	0.944
Kohl	295.400^b^	1.633	3	0.652
Lipstick	296.044^b^	2.277	3	0.517
Sindoor	302.347^b^	8.581	3	0.035
Surma	294.065^b^	0.298	3	0.960
Utensils	333.493^b^	39.727	12	**0.000**
FuelSource	307.054^b^	13.287	6	**0.039**
NonSpecific	339.528^b^	45.761	15	**0.000**
GastroIntestinal	305.094^b^	11.327	9	0.254
PiccaSymptoms	381.297^b^	87.530	6	**0.000**

Bold values indicate *p*-value < 0.05

^a^This reduced model is equivalent to the final model because omitting the effect does not increase the degrees of freedom

^b^Unexpected singularities in the Hessian matrix are encountered. This indicates that either some predictor variables should be excluded or some categories should be merged

### Boruta algorithm

A feature might be important in the prediction of an outcome, and yet may not be captured in the analysis. For example, the logistic regression method used in [32, 41], to carry out the multivariate analysis, may not capture all the features that are important. This is because the logistic regression captures only certain relationships (linear) between the input and output variables. Therefore, here we use another feature selection method called Boruta. The Boruta algorithm is statistically grounded and works well without any specific input from the user [44]. It was developed as a package for R and operates as a wrapper algorithm around the Random Forest. The algorithm iteratively removes features that are proven to be statistically less relevant [44]. The Boruta algorithm is applied to the 18-feature set and from this selection method, 12 features are found to be useful (Table 8). From the Boruta application, we get a more detailed number of features that are significant in the prediction of the *lead content level*.

**Table 8:** Significant features obtained by applying the Boruta algorithm to the four-label dataset

Age	TakeHomeExposure	Lipstick
WaterSource	OccupationalExposure	Sindoor
NonSpecific	Educ_Categorised	Utensils
GastroIntestinal	PiccaSymptoms	Kohl

### Evaluation of prediction models

To test the built prediction models using a set of classification algorithms described earlier, evaluation is performed for different scenarios. The models are built with different feature sets obtained by applying univariate and multivariate analysis. The prediction accuracy is used as a performance index to evaluate the performance of each of the built classifiers. Due to an imbalance in the data, there is a chance that the model built has a bias towards for the majority class, causing a greater prediction error for the minority class. On the other hand, if the model fails to identify potential lead risks, in such scenarios the subject misses the subsequent benefits from early screening and treatment. In turn, there will be financial implications. Therefore, the evaluation is also carried out on the balanced dataset and compared against the imbalanced data.

### Model prediction performance

**Fig. 13:**
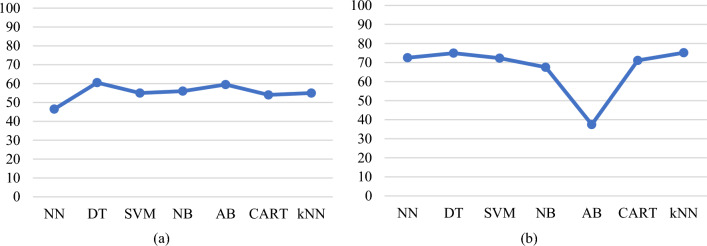
**a** Average prediction accuracies of the models obtained by applying cross-validation and built with 18 feature sets and original dataset (Four class labels), and **b** average prediction accuracies of the models obtained by applying cross-validation and built with 18 feature set and rebalanced dataset (Four class labels)

In the first scenario, models are built using the original data without handling the data imbalance. The prediction accuracies are compared between models built using different classification algorithms, on the given dataset with the 18 features and four class labels (ND_5, Between5_10, Between10_15, and GreaterThan15). A 10-fold cross-validation test with 10 iterations is performed to find the relationship between the classifier accuracy and features. Figure 13a presents a plot of average prediction accuracies for different models built using 18 feature sets learned and tested on the original data with four class labels. Next, the models are built using the resampled data, following the resampling process described earlier. The resampled data consists of 105 instances of each class of ND_5, Between5_10, Between10_15, and GreaterThan15. Figure 13b presents a plot of average prediction accuracies for different models and 18 feature sets learned and tested on the rebalanced data with four class labels. As can be seen from Fig. 13a, b, rebalancing has not increased the accuracy of the built model in all the cases. However, the balancing allows the model to perform the class labelling of an unknown observation solely based on the observed values of the selected features and the robustness of the classification algorithms applied.

**Fig. 14:**
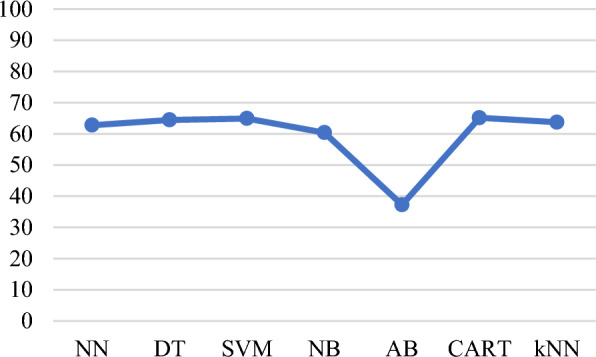
Average prediction accuracies of the models obtained by applying a cross-validation test built with five features obtained from the Chi-square test (balanced and four class labels dataset)

Next, the models are built with features obtained from the univariate analysis. A set of models are built with five features obtained from the Chi-Square test. Figure 14 shows the average prediction accuracy obtained from the cross-validation test for the models built with five features and a balanced dataset (Four class labels). The classification results shown in Figs. 13a and 14 show that NN and *k*NN have a better performance in comparison to the other built models on the balanced dataset.

**Fig. 15:**
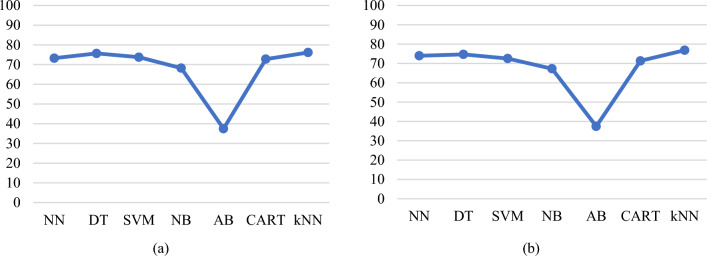
**a** Average prediction accuracies of the models obtained by applying a cross-validation test built with 8 features obtained from multinomial logistic regression (Balanced and four class labels dataset), and **b** average prediction accuracies of the models were obtained by applying a cross-validation test built with 12 features obtained from the Boruta algorithm (Balanced and four class labels dataset)

Next, the models are built using the features obtained from multivariate analysis. Figure 15a presents a plot of average prediction accuracies for different models built with eight features obtained from multinomial logistic regression. Figure 15b presents a plot of average prediction accuracies for different models built with 12 features obtained from the Boruta algorithm. From the classification results shown in Fig. 15a, b, the models built with the features obtained from the Boruta algorithm have a slightly improved accuracy, particularly for NN and *k*NN models.

**Fig. 16:**
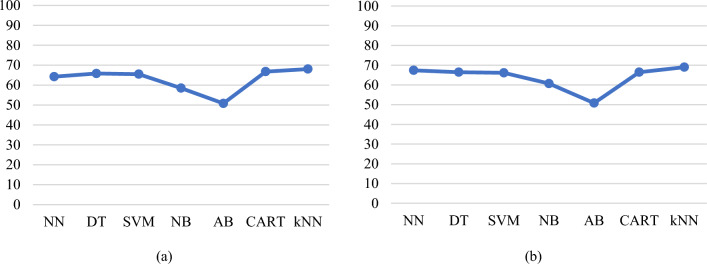
**a** Average prediction accuracies of the models obtained by applying a cross-validation test built with 18 features (Balanced and three class labels dataset). **b** Average prediction accuracies of the models were obtained by applying a cross-validation test built with 11 features obtained from the Boruta algorithm (Balanced and three class labels dataset)

Lead toxicity greater than 10 μg/dL is still considered high and different countries have different regulations on the upper limit of lead levels. Therefore, it would be ideal to provide models with two upper ranges of lead toxicity, one as GreaterThan15 and the other one as GreaterThan10. The data points where the BLL is between 10 and 15 μg/dL and greater than 15 μg/dL are merged to form one class. The new class labels are ND_5 (105), Between5_10 (52), and GreaterThan10 (43). SMOTE is applied to this dataset for balancing. Following this, the Boruta algorithm is applied to the three output class data resulting in 11 features, which includes all the features shown in Table 8, excluding Lipstick. Figure 16a presents a plot of average prediction accuracies for different models built with 18 features obtained with three class labels. Figure 16b presents a plot of average prediction accuracies for different models built with 11 features obtained from the Boruta algorithm.

## Discussion

In this paper, we analysed various sources of lead exposure in pregnant women, addressed the possibility of accurately predicting lead toxicity levels based on the identified set of features, and analysed what features support or do not support prediction. Features collected in this dataset are significantly more relevant and detailed. The analysis established a correlation between specific features and higher lead concentration with a certain degree of certainty. Following a data-driven approach, we used ML algorithms to build lead prediction models, explored patterns in the collected dataset, balanced the dataset, and identified a set of sociodemographic features that can model lead toxicity. We also investigated the significance of reducing the feature set, and categorisation of features into fewer sub-categories. The initial set of models was built with an 18 features dataset. In comparison to the unbalanced dataset, the rebalanced dataset had a higher accuracy for all the cases of the built models (Fig. 13a, b). From the existing literature, we know that all the features collected in the dataset contribute to lead toxicity. However, what we do not know is to what extent each of these features was useful in determining lead toxicity and which features were noisy. It is crucial that only those features which are deemed to be important are included in the model and therefore, feature selection is carried out. This enables reduced complexity and computational cost, faster training, easy-to-interpret models, improved accuracy, and minimised overfitting. To produce more reliable models that are accurate in prediction, feature selection is done followed by exploratory data analysis to find the best prediction models with an optimal set of features. A univariate analysis is carried out initially and the results suggested that a univariate approach may not succeed in identifying all the useful or relevant features. Therefore, next, we used multivariate feature selection methods in our work. Multinomial logistic regression and the Boruta algorithm were applied, and a set of more detailed and relevant features were identified. The 8-feature models obtained from the multinomial logistic regression analysis achieved improved accuracy (*k*NN = 76.13%, DT = 75.66%, and NN = 73.27% for a cross-validation test). In comparison to the models built with the features obtained from the multinomial logistic regression, the models built with 12 features from the Boruta algorithm achieved slightly higher accuracy (*k*NN = 76.84%, DT = 74.70%, and NN = 73.99% for a cross-validation test).

One of the challenges for the ML-based prediction models is wider acceptability and adoption in the current healthcare systems. The built models are required to demonstrate transparency and justify the rationale behind the prediction. The end user of the built lead prediction models is healthcare professionals with a non-technical background in general. As a result, it is essential that the built models are easy to analyse and interpret and therefore, ease of use and outcomes are the key features for these kinds of healthcare-based applications. DTs are considered valuable to healthcare-based applications as the decision-making is transparent and can be visualised as trees; thus, making easy analysis and interpretation [41]. DT allows the outcomes to be clinically understood and rationalised, keeping in mind that healthcare professionals have a non-technical background. On the other hand, the *k*NN-based models are based on finding the nearest neighbour for an unknown case using the similarity measure between the unknown case and its neighbours. This characteristic of *k*NN is useful for healthcare professionals. Based on the observed values for an unknown case, the class label from the prediction model can be interpreted by the healthcare professional based on their previous experience with a similar set of values observed for lead toxicity. Contrary to the *k*NN, the output from more complex models such as NNs and SVMs is a challenging job for non-technical professionals [41].

Given the budget and limited data collected, we built a proof of concept to demonstrate the extendibility of ML approaches to build a lead prediction framework. Previous works available in the existing literature have collected data with the aim of establishing a correlation between sociodemographic features and lead toxicity. However, they haven’t made this data actionable and used ML and advanced analytics to deliver more valuable information at the point of care. Instead of clinically reviewing every pregnant woman for lead toxicity exposure and referring them to lab-based testing, subjects could be screened using the ML-based lead prediction model. The models can assist in detecting early indicators of lead toxicity prediction. Even though the accuracy obtained in the work might be low due to limited data, it demonstrated the possibility of using non-invasive, easy-to-collect sociodemographic features along with computational modelling to predict the lead toxicity level with a certain degree of certainty (reflected in the prediction performance). Backed up by more data, in future, the work can be extended to provide greater speed and precision along with insights that can help healthcare providers plan and deliver care in the context of lead poisoning.

## Conclusion and future work

Data-driven decisions increasingly make the difference and built models can help in avoiding unknown risks of lead toxicity. Doing an in-depth analysis of various reasons for lead exposure and identifying possible features pointing to lead exposure in pregnant women is meaningful. With the limited data, we were able to demonstrate the possibility of using ML techniques for predicting lead toxicity levels. The current work describes the initial framework for lead prediction modelling that can underpin, in future, an analytical tool in the form of a mobile screening app with a more detailed set of data and improved accuracy. Currently, more data is being collected to validate the build prediction model and, design and development of the screening app.

The proposed work is novel in establishing a truly interdisciplinary work and utilising the benefits of mobile technology and ML to reach out to a larger population. The approach of modelling in the area of lead poisoning can be significant, where the prediction of lead level can be made based on the observed features without using expensive analytical instruments. In future, the proposed work can bring a positive impact on survival, growth, and development along with a constructive informed healthcare system. The screening app can be used as a referral system to meet a medical expert for further investigation and early intervention. Advice aimed at reducing lead toxicity can be beneficial. For example, in the US to reduce lead toxicity it is advised that the intake of foods rich in vitamin C, iron and calcium diet can influence the overall lead burden in two crucial ways: (1) through the lead content of particular food items, and (2) by the alteration of lead assimilation and excretion by individual nutrients and by the physiochemical properties of the food [45]. It has been observed that iron and calcium intakes seem to be inversely associated with blood lead concentrations in pregnancy [11].

In future, with more robust prediction models, the pressure on the overstretched healthcare systems, in developing countries, can be released by utilising the facilities that can be provided through health centres, with the help of healthcare workers who are not necessarily experts. If a high exposure is detected, pre-emptive measures can be taken to minimise the effects of lead exposure. The early detection and prevention of lead-related risk levels will help in a lower percentage of low-birth-weight babies, enhanced health facilities, and lower infant and mother mortality rates. A public health intervention of this kind would be able to inform and educate the population about these yet unknown sources of lead intoxication. The finding of a significant proportion will help in developing new protocols for screening, developing a strategy of prevention, and building constructive informed healthcare policies. Such findings will be beneficial in providing fast reliable social services, quality, and affordable health to mothers. Consequently, it will increase the strength of the national workforce, and thus increase the economy of the countries. Additionally, it can reduce the costs that governments incur on medical and healthcare support expenditures. Significantly, our proposed research could help to attain the UN’s 2030 Sustainable Development Goals (SDGs) 3 (‘good health and well-being’) and 10 (‘reduced inequalities’) [46].

## Data Availability

The data sharing is not possible as per the ethics reviewed.

## References

[1] Rocha A, Trujillo KA (2019). Neurotoxicity of low-level lead exposure: history, mechanisms of action, and behavioural effects in humans and preclinical models. Neurotoxicology.

[2] Roberts DJ, Bradberry SM, Butcher F, Busby A (2022). Lead exposure in children. BMJ.

[3] WHO. Lead poisoning. World Health Organisation; 2023. https://www.who.int/news-room/fact-sheets/detail/lead-poisoning-and-health. Accessed 5 Oct 2023.

[4] Taylor CM, Doerner R, Northstone K, Kordas K (2019). Dietary patterns are not consistently associated with variability in blood lead concentrations in pregnant British women. J Nutr.

[5] Whitehead LS, Buchanan SD (2019). Childhood lead poisoning: a perpetual environmental justice issue?. J Public Health Manag Pract.

[6] Ericson B, Hu H, Nash E, Ferraro G, Sinitsky J, Taylor MP (2021). Blood lead levels in low-income and middle-income countries: a systematic review. Lancet Planet Health.

[7] Grantham-McGregor S, Cheung YB, Cueto S, Glewwe P, Richter L, Strupp B (2007). Developmental potential in the first 5 years for children in developing countries. Lancet.

[8] Rees N, Fuller R. The toxic truth: children’s exposure to lead pollution undermines a generation of future potential. UNICEF; 2020.

[9] Wright JP, Dietrich KN, Ris MD, Hornung RW, Wessel SD, Lanphear BP, Rae MN (2008). Association of prenatal and childhood blood lead concentrations with criminal arrests in early adulthood. PLoS Med.

[10] Aizer A, Currie J (2019). Lead and juvenile delinquency: new evidence from linked birth, school, and juvenile detention records. Rev Econ Stat.

[11] Taylor CM, Golding J, Hibbeln J, Emond AM (2013). Environmental factors predicting blood lead levels in pregnant women in the UK: the ALSPAC study. PLoS ONE.

[12] Rudge CV, Röllin HB, Nogueira CM, Thomassen Y, Rudge MC, Odland JØ (2009). The placenta as a barrier for toxic and essential elements in paired maternal and cord blood samples of South African delivering women. J Environ Monit.

[13] Schell LM, Denham M, Stark AD, Gomez M, Ravenscroft J, Parsons PJ, Samelson R (2003). Maternal blood lead concentration, diet during pregnancy, and anthropometry predict neonatal blood lead in a socioeconomically disadvantaged population. Environ Health Perspect.

[14] Lanphear BP, Rauch S, Auinger P, Allen RW, Hornung RW (2018). Low-level lead exposure and mortality in US adults: a population-based cohort study. Lancet Public Health.

[15] Amadi CN, Igweze ZN, Orisakwe OE (2017). Heavy metals in miscarriages and stillbirths in developing nations. Middle East Fertil Soc J.

[16] Sanders AP, Svensson K, Gennings C, Burris HH, Oken E, Amarasiriwardena C, Tellez-Rojo MM (2018). Prenatal lead exposure modifies the effect of shorter gestation on increased blood pressure in children. Environ Int.

[17] Poropat AE, Laidlaw MA, Lanphear B, Ball A, Mielke HW (2018). Blood lead and preeclampsia: a meta-analysis and review of implications. Environ Res.

[18] Hanna-Attisha M, Lanphear B, Landrigan P (2018). Lead poisoning in the 21st century: the silent epidemic continues. Am J Public Health.

[19] Gould E (2009). Childhood lead poisoning: conservative estimates of the social and economic benefits of lead hazard control. Environ Health Perspect.

[20] Satyanarayana VA, Jackson C, Siddiqi K, Chandra SP, Huque R, Dherani M, Nasreen S, Murthy P, Rahman A (2021). A behaviour change intervention to reduce home exposure to second-hand smoke during pregnancy in India and Bangladesh: a theory and evidence-based approach to development. Pilot Feasibility Stud.

[21] Kumar S, Sharma A, Kshetrimayum C (2019). Environmental and occupational exposure and female reproductive dysfunction. Indian J Med Res.

[22] Levallois P, Barn P, Valcke M, Gauvin D, Kosatsky T (2018). Public health consequences of lead in drinking water. Curr Environ Health Rep.

[23] Lewis J (2022). True colors: unmasking hidden lead in cosmetics from low- and middle-income countries. Environ Health Perspect.

[24] Chaudhary S, Firdaus U, Ali SM, Mahdi AA (2018). Factors associated with elevated blood lead levels in children. Indian Pediatr.

[25] Tanaka S, Teshima K, Verhoogen E (2022). North-South displacement effects of environmental regulation: the case of battery recycling. Am Econ Rev Insights.

[26] Grossman D, Slutsky DJ. The effect of an increase in lead in the water system on fertility and birth outcomes: the case of Flint, Michigan (2017).

[27] Shadab GHA, Afzal M (2021). Lead and zinc interactions—an influence of zinc over lead related toxic manifestations. J Trace Elem Med Biol.

[28] Słota M, Wąsik M, Stołtny T, Machoń-Grecka A, Kasperczyk S (2022). Effects of environmental and occupational lead toxicity and its association with iron metabolism. Toxicol Appl Pharmacol.

[29] Hauptman M, Stierman B, Woolf AD (2019). Children with autism spectrum disorder and lead poisoning: diagnostic challenges and management complexities. Clin Paediatr.

[30] Ansari JA, Ahmad MK, Verma AK, Fatima N, Jilani H (2015). Microwave assisted determination of minerals and toxic metals in traditionally used medicinal plant *Zingiber officinale* Roscoe by inductively coupled plasma-optical emission spectrometer. Int J Adv Res.

[31] Krampitz P, Smith S. Increased laboratory productivity for ICP-OES applied to US EPA method 6010C. https://resources.perkinelmer.com/corporate/cmsresources/images/46-74168app_icp-oesappliedtoepamethod6010c.pdf. Accessed 18 July 2023.

[32] Chaurasia P, McClean SI, Nugent CD, Cleland I, Zhang S, Donnelly MP, Tschanz J (2016). Modelling assistive technology adoption for people with dementia. J Biomed Inform.

[33] Shabani M, Hadeiy SK, Parhizgar P, Zamani N, Mehrad H, Hassanian-Moghaddam H, Phillips S (2020). Lead poisoning; a neglected potential diagnosis in abdominal pain. BMC Gastroenterol.

[34] Kumar A, Kumar A, Cabral-Pinto MMS, Chaturvedi AK, Shabnam AA, Subrahmanyam G, Yadav KK (2020). Lead toxicity: health hazards, influence on food chain, and sustainable remediation approaches. Int J Environ Res Public Health.

[35] Tiwari AKM, Mahdi AA, Mishra S, Parveen H, Fatima G (2020). Effect of iron and folate supplementation on Pb levels in pregnant anaemic women: a prospective study. Free Radic Res.

[36] Shaffer RM, Gilbert SG (2018). Reducing occupational lead exposures: strengthened standards for a healthy workforce. Neurotoxicology.

[37] World Health Organization. Preventing disease through healthy environments: exposure to lead: a major public health concern. WHO; 2023.

[38] Ettinger AS, Leonard ML, Mason J (2019). CDC’s lead poisoning prevention program: a long-standing responsibility and commitment to protect children from lead exposure. J Public Health Manag Pract.

[39] Chaurasia P, McClean S, Scotney B, Nugent C (2012). Duration discretisation for activity recognition. Technol Health Care.

[40] Wu X, Kumar V, Quinlan JR, Ghosh J, Yang Q, Motoda H, Steinberg D (2007). Top 10 algorithms in data mining. Knowl Inf Syst.

[41] Chaurasia P, McClean S, Nugent CD, Cleland I, Zhang S, Donnelly MP, Tschanz J (2022). Modelling mobile-based technology adoption among people with dementia. Pers Ubiquitous Comput.

[42] Pears R, Finlay J, Connor AM. Synthetic Minority over-sampling technique (SMOTE) for predicting software build outcomes. 2014. arXiv preprint arXiv:1407.2330.

[43] Böhning D (1992). Multinomial logistic regression algorithm. Ann Inst Stat Math.

[44] Kursa MB, Rudnicki WR (2010). Feature selection with the Boruta package. J Stat Softw.

[45] Kordas K (2017). The “lead diet”: can dietary approaches prevent or treat lead exposure?. J Paediatr.

[46] Weiland S, Hickmann T, Lederer M, Marquardt J, Schwindenhammer S (2021). The 2030 agenda for sustainable development: transformative change through the sustainable development goals?. Polit Gov.

